# Splice Isoforms of the Polyglutamine Disease Protein Ataxin-3 Exhibit Similar Enzymatic yet Different Aggregation Properties

**DOI:** 10.1371/journal.pone.0013695

**Published:** 2010-10-27

**Authors:** Ginny Marie Harris, Katerina Dodelzon, Lijie Gong, Pedro Gonzalez-Alegre, Henry L. Paulson

**Affiliations:** 1 Graduate Program in Molecular and Cellular Biology and Medical Scientist Training Program, University of Iowa, Iowa City, Iowa, United States of America; 2 University of Iowa Carver College of Medicine, Iowa City, Iowa, United States of America; 3 Department of Neurology, University of Michigan, Ann Arbor, Michigan, United States of America; 4 Department of Neurology, University of Iowa, Iowa City, Iowa, United States of America; National Institutes of Health, United States of America

## Abstract

Protein context clearly influences neurotoxicity in polyglutamine diseases, but the contribution of alternative splicing to this phenomenon has rarely been investigated. Ataxin-3, a deubiquitinating enzyme and the disease protein in SCA3, is alternatively spliced to encode either a C-terminal hydrophobic stretch or a third ubiquitin interacting motif (termed 2UIM and 3UIM isoforms, respectively). In light of emerging insights into ataxin-3 function, we examined the significance of this splice variation. We confirmed neural expression of several minor 5′ variants and both of the known 3′ ataxin-3 splice variants. Regardless of polyglutamine expansion, 3UIM ataxin-3 is the predominant isoform in brain. Although 2UIM and 3UIM ataxin-3 display similar *in vitro* deubiquitinating activity, 2UIM ataxin-3 is more prone to aggregate and more rapidly degraded by the proteasome. Our data demonstrate how alternative splicing of sequences distinct from the trinucleotide repeat can alter properties of the encoded polyglutamine disease protein and thereby perhaps contribute to selective neurotoxicity.

## Introduction

The polyglutamine neurodegenerative diseases are caused by the expansion of polyglutamine-encoding CAG trinucleotide repeats within specific genes. Polyglutamine expansion promotes disease protein misfolding, triggering a pathogenic cascade leading to neurodegeneration, with age of disease onset inversely correlated to expansion length. While all polyglutamine disease proteins are widely expressed, the patterns of neurodegeneration and clinical manifestations of disease vary significantly [1]. This variability suggests that the protein context of each expansion contributes to selective neuronal toxicity by influencing factors such as subcellular localization, protein-protein interactions, endogenous function, and aggregation. Cell-specific elements of protein context are particularly attractive candidate determinants of selective toxicity. Because the precise protein context of a disease protein will vary between splice isoforms, alternative splicing may influence patterns of polyglutamine protein-induced neurodegeneration.

Alternative splicing is an important mechanism by which proteomic diversity is achieved in eukaryotes, with most mammalian genes encoding more than one transcript variant [2], [3]. Patterns of alternative splicing can be cell-specific and regulated through physiological or pathological processes. Many transcripts that encode polyglutamine proteins are alternatively spliced [4]–[15]. For example in SCA6, splice variants encoding the polyglutamine domain of the Cav2.1 calcium channel are specifically enriched in Purkinje cells of SCA6 patients but not in controls [4]. This finding underscores the possibility that alternative splicing contributes to polyglutamine disease pathogenesis by regulating the expression of more “toxic” transcripts in specific cell populations.

**Figure 1:**
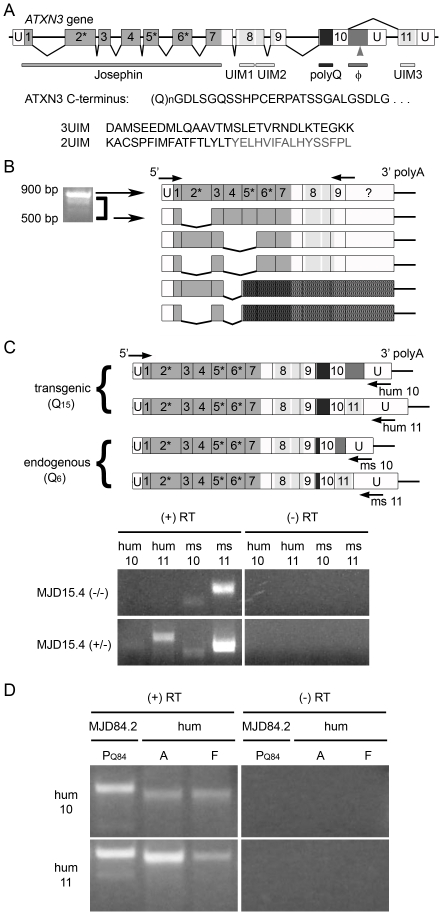
Ataxin-3 is alternatively spliced in *ATXN3* YAC transgenic and human brain. (A) Schematic representation of the *ATXN3* gene showing exons that encode specific functional domains. Untranslated regions (U) are not drawn to scale. The splicing pattern of the originally identified 2UIM ataxin-3 transcript is shown below, while above is shown the alternative splicing that links exon 10 to exon 11, generating 3UIM ataxin-3. Asterisks indicate exons that encode amino acids comprising the catalytic triad, polyQ denotes the polyglutamine domain, and the arrowhead indicates a polymorphic Tyr/Stop-encoding residue within the hydrophobic domain (Φ) of the C-terminus of 2UIM ataxin-3. C-terminal amino acid sequences are shown below the diagram, beginning with shared sequence in both isoforms extending from the polyQ domain, followed by the divergent sequences for the 2UIM and 3UIM isoforms; residues omitted in some SNP variants of the 2UIM isoform are shown in grey. (B) Diagram showing 5′ ataxin-3 splice variants identified and confirmed by sequencing. Multiple variants are detectable in mature mRNA from adult murine brain (and fetal brain, data not shown) by RT-PCR, using primers targeting the 5′UTR/exon 1 junction and exon 9 (arrows). All identified splice variants that maintain the open reading frame eliminate at least one catalytic triad residue, and thus are not likely to encode an active DUB. Darkly shaded areas are downstream of a frameshift-induced stop codon. (C–D) Endogenous *Atxn3* and transgenic *ATXN3* “full length” splice variants were amplified by RT-PCR using species-specific (human, hum; murine, ms) and sequence-specific (10-exon 2UIM-encoding, 10; 11-exon 3UIM-encoding, 11) primers. 10-exon and 11-exon variants are both detectable in mature mRNA: (C) endogenous *Atxn3* from all murine samples and unexpanded *ATXN3* from MJD15.4(+/−) brain; and (D) expanded *ATXN3* from MJD84.2(+/−) brain, and unexpanded *ATXN3* from pooled human brain tissue (hum). Perinatal day 1–3 (P_Q84_), adult (A), or fetal (F) sources were used, as indicated. Note: Primers are not drawn to scale; see Materials and Methods for exact sequences and locations.

Here we explore alternative splicing in the polyglutamine disorder Spinocerebellar ataxia type 3 (SCA3), the most common dominantly inherited ataxia. The disease protein in SCA3, ataxin-3, is a deubiquitinating enzyme [16]–[19]. The original ataxin-3 transcript isolated from human brain encodes an isoform that contains a Josephin protease domain, two ubiquitin interacting motifs (UIMs), and the polyglutamine domain, followed by a C-terminal stretch of hydrophobic amino acids [20]. Goto and colleagues subsequently isolated a variant that encodes a third UIM at its C-terminus instead of this hydrophobic tail [11]. The protein products of these alternative splice variants are termed the 2UIM and 3UIM ataxin-3 isoforms, respectively (Figure 1A). The 3UIM isoform is known to be widely expressed [21]. Additional N-terminal splice variants have also been observed in human and rodent tissues [8], [9], [13]. Although 2UIM and 3UIM ataxin-3 are both considered “full length” ataxin-3 isoforms, and both have been used in mechanistic studies of SCA3, the impact of this 3′ splice variation on ataxin-3 function and disease pathogenesis has not been examined.

In the current study, we investigate ataxin-3 alternative splicing. We characterize the range of splice variation in human and transgenic murine brain, establishing that while both 3′ splice variants are expressed, 3UIM ataxin-3 is the predominant isoform. We further show that although C-terminal splice isoform variation does not influence ataxin-3′s deubiquitinating activity, it significantly modifies both its tendency to aggregate and its intracellular stability. This observation highlights how splicing events that preserve the polyglutamine domain but alter protein context could influence selective neuronal toxicity.

## Materials and Methods

*Animal lines* – Three murine models of SCA3 were used in this study. MJD15.4 and MJD84.2 [22] are yeast artificial chromosome (YAC) transgenic lines that contain the full human *ATXN3* gene with an unexpanded (Q_15_-encoding) or expanded (Q_84_-encoding) repeat. The presence of all genomic elements of the *ATXN3* gene allows these mice to exhibit alternative splicing of both transgenic (human) and endogenous (murine) ataxin-3. Both lines possess the Tyr-encoding version of an A/C SNP within the extended portion of exon 10, at the position that encodes the final stop codon seen in the MJD1a isoform (GenBank accession no. **S75313.1**), resulting in 2UIM-long isoforms that share an extreme C-terminus (though not all SNPs) with isoform MJD2-1 [11]. Q71-B transgenic mice [23] express the human MJD1a splice isoform as a cDNA driven by the prion promoter, and thus only exhibit alternative splicing of endogenous ataxin-3. *Atxn-3* knockout mice [19] and their littermate controls were used to confirm that ataxin-3 isoforms detected in various tissues by 1H9 mAb were in fact derived from the *Atxn3* gene. All lines were maintained in accordance with the University of Michigan and University of Iowa AUCUC guidelines, including accepted measures to minimize pain or discomfort.

### Constructs and Primers

3UIM (“full length, FL”) and catalytically inactive C14A GST-ataxin-3 fusion proteins were derived from pGEX-6P-1, as previously described [24]. The 3UIM-encoding pGEX6P1-At3(Q22)FL expression vector has also been previously described as pGEX6P1-ATX3-WT [16]. pGEX6P1-At3(Q22)2UIM was derived from this construct by substituting the region downstream of the (CAG) repeat in the 3UIM-encoding construct for the 2UIM-encoding sequence in pEGFP-C1-ataxin-3(Q28) [25]. Briefly, Q28 ataxin-3 was amplified using MJD.Nter.F#722 (see below) and the primer hMJD2UIM-R1N (5′gcggccgctcttatgtcagataaagtg 3′), which creates a novel NotI restriction site, cloned into pCR2.1-TOPO (Invitrogen, Cat # K4500-01), and restriction digested from an endogenous PpuMI site downstream of the repeat to the novel NotI site to generate the donor 3′ sequence. The N-terminally Flag-tagged ataxin-3 eukaryotic expression vector pFlag-A22-FL-M1G contains the full 3UIM-encoding ataxin-3 sequence in a pFlag-CMV-6a backbone. pFlag-A22-2UIM-M1G was generated by inserting the 2UIM-encoding 3′ region, as described above. The pFlag-A22-UIM3(SA/DG)-M1G mutant was similarly generated by exchanging regions of pFlag-A22-FL-M1G and pGEX6P1-At3(Q22)UIM3(SA/DG) using endogenous ataxin-3 MfeI and vector-derived NotI sites. All constructs were confirmed by restriction digestion and DNA sequencing.

MJD.Nter.F#722 (5′ ataaacatggagtccatcttc 3′) was the common forward primer used to amplify human and murine ataxin-3 cDNA. This primer targets the junction of the 5′UTR and exon1. The following reverse primers were used to amplify transgenic 5′ splice variants from YAC cDNA: HuMJD.Cter1.R#724 (5′ gtgtcatatcttgagatatg 3′) and HuMJD.Cter2.R#723 (5′ ttctgaagtaagatttgtac 3′) target exon 9 of human ataxin-3 to amplify 5′ variants independently of the documented 3′ variation. The following reverse primers were used to amplify 3′ splice variants from cDNA pools: 2HumExon10R#537 (5′ ctgctccttaatccagg 3′) was used to amplify transgenic and endogenous human 10-exon specific transcripts, 2HumExon11R#536 (5′ cacacggtatacagttgaagg 3′) was used to amplify transgenic and endogenous human 11-exon specific transcripts, MuMJDexon10R#598 (5′ cgagtaaagcatcactg 3′) was used to amplify endogenous murine 10-exon specific transcripts, and MuMJDexon11R#597 (5′ ctgactgcctctttggc 3′) was used to amplify endogenous murine 11-exon specific transcripts.

### Cell culture and transfection

Cos7 and 293T cells were maintained at 37°C, 5% CO_2_ in DMEM, 10% FBS, 1% penicillin/streptomycin. Cells were transiently transfected with 0.2–2.0 µg DNA, using Lipofectamine PLUS Reagents (Invitrogen Cat #18324012, #11514-015), as per the manufacturer's recommendations. Mock transfections were performed without DNA, and vector control transfections were performed with equivalent amounts of empty vector DNA.

### Collection of animal tissues

Adult mice were either euthanized with Ketamine/Xylazine (1 mg/0.1 mg per gram) prior to immediate dissection of brain tissue, or deeply anesthetized with 0.2 mg Ket/20 µg Xyl per gram prior to transcardial perfusion (2 ml per minute) with 5–10 ml of ice cold sterile PBS followed by dissection of brain and other tissues. Neonatal mice were euthanized by decapitation. Harvested tissue was either homogenized immediately, snap frozen, or prepared for archival storage using RNAlater (Ambion, Cat #AM7020).

### Preparation of cDNA from animal tissues and pooled human RNAs

Total RNA was isolated from murine tissues using TRIzol Reagent (Invitrogen, Cat #15596-026), and mature mRNA was extracted from total RNA using the Poly(A)Purist kit (Ambion, Cat #1916) as per the manufacturers' standard protocols. Adult and fetal human total RNA were purchased from Clontech: the adult RNA (Cat # 636530, Lot 7120601A) was pooled from two neurologically normal male Caucasians, aged 47–55, the fetal RNA (Cat # 636526, Lot 7080344) was pooled from 21 third trimester spontaneous abortions, gestational age 26–40 weeks. Total cDNA was generated using random hexamer priming of mature mRNA with Superscript III Reverse Transcriptase (Invitrogen, Cat # 18080-085), prior to transcript-specific analyses.

### Sequence specific PCR-amplification of cDNA

All 5′ and 3′ splice variants were amplified from reverse-transcribed cDNA using the following conditions: 94°C, 2 min; (94°C, 30 s; 50°C, 30 s; 72°C, 1 min)×35 cycles; 72°C, 1–10 min; 4°C, until further analysis. Fifty microliter reactions were carried out with Taq polymerase (Invitrogen, Cat # 10342-053) or Platinum Taq High Fidelity polymerase mixture (Invitrogen, Cat # 11304-011), following the manufacturer's protocols in the recommended buffer systems, using 2 µl cDNA template.

### Preparation of protein from murine tissues and cultured cells

For splice isoform analysis in tissue homogenates, protein was isolated in conjunction with total RNA, using the TRIzol Reagent, as per the manufacturer's recommendations. For ataxin-3 aggregation analysis and 2D-PAGE, non-denaturing whole brain lysates were prepared by homogenization of 100 mg tissue per milliliter of ice cold RIPA buffer (50 mM Tris-HCl, pH 7.4, 150 mM NaCl, 1% NP-40, 0.5% sodium deoxycholate, 0.1% SDS) plus Complete Mini Protease Inhibitor Cocktail (PI) (Roche, Cat# 11836153001) in a Potter-Elvehjem homogenizer, and then centrifuged at 4000 rpm, 15 min, 4°C to separate supernatant and pellet fractions. Pellet fractions were re-homogenized in an equal volume of RIPA+PI. All RIPA lysates were stored at −80°C. Lysates were diluted in Laemmli buffer (50 mM Tris-Cl, pH 6.8, 2% SDS, 10% glycerol, 0.1% bromophenol blue) plus 100 mM DTT and sonicated 5–10 s for SDS-PAGE followed by Western blot analysis utilizing Western Lightening ECL (Perkin-Elmer Life Sciences, Cat # NEL102001EA) or dual color visualization of IRDye conjugated secondary antibodies (LI-COR Biosciences, Cat # 926-32210 & 926-32221) on an Odyssey IR imaging system. Densitometry for aggregate analysis was carried out using ImageJ software, after background subtraction with a rolling ball radius of 50; soluble and insoluble ataxin-3 levels were normalized to endogenous murine ataxin-3 signal to control for equal protein loading.

For transient transfection experiments, adherent cells were washed once with ice cold PBS, then directly lysed in Laemmli buffer plus 100 mM DTT (440 µl/well for 6-well plates; 200 µl/well for 12-well plates), sonicated for 5–10 s, heated for 3 minutes at 95°C, and centrifuged for 3 minutes at 14,000 rpm prior to SDS-PAGE and Western blot analysis, as above.

### 2D-Western Blot Analysis

Non-denaturing whole brain RIPA lysates (50 µg total protein) or purified GST-ataxin-3 proteins (5 µg for in gel Coomassie detection, 50 ng for Western analysis) were diluted into FOSB1 (7 M urea, 2 M thiourea, 1.25% CHAPS 32 mM DTT, 2.5 mM TCEP, 0.5% ASB-14, 0.5% Triton X-100, 0.5% Zwittergent 3–10, 0.3% carrier ampholytes, 0.001% Bromophenol Blue) using 100x BioLytes 3.9–5.1 as the carrier ampholytes (BioRad, Cat # 1632098) for optimal narrow range resolution. Diluted samples were used to passively rehydrate 11 cm narrow range pH 3.9–5.1 immobilized pH gradient (IPG) strips (BioRad, Cat # 163-2024). Isoelectric focusing was carried out in a PROTEAN IEF cell under the following conditions: Step 1 (0–250 V, 15 min, rapid ramp), Step2 (250–8000 V, 1 hr, slow ramp), Step 3 (8000 V constant, 30,000 V-hr, rapid ramp); all steps were set to a default temperature of 20°C, and wicks were changed periodically to remove unwanted salts and enhance actual running time. Focused IPG strips were equilibrated, run in the second dimension, and transferred to PVDF membrane using the Criterion Blotter system, as per the manufacturer's recommendations.

### *In vitro* deubiquitination assays

Recombinant 2UIM, 3UIM, and C14A ataxin-3(Q22) were expressed in BL21-A1 *E. coli* (Invitrogen Cat #C607003) as GST fusion proteins and purified as follows. Overnight cultures were subcultured at 1 ml per 100 ml LB +50 µg/ml Ampicillin until an OD_600_ between 0.4–0.6. Recombinant protein expression was then induced with 400 µM isopropyl-1-thio-β-D-galactopyranoside for 3 hr at 30°C. Bacteria were lysed by sonication in 0.5x NPG buffer (150 mM NaCl, 25 mM NaH_2_PO_4_, 5% glycerol, pH 8.0) plus protease inhibitors (1x Sigma Protease Inhibitor Cocktail, 1x Roche Complete mini Protease Inhibitor Cocktail, 2.25 mM PMSF, 0.5 mM Peflabloc SC). Lysates were pre-cleared for 15 minutes in 0.5xNPG-equilibrated PABA-agarose, and recombinant proteins were then bound to equilibrated GST-Sepharose, (GE Healthcare, Cat #27-4570-03) for 30 minutes, on ice with periodic mixing, washed 5x with PBS plus Roche Complete Mini Protease Inhibitor Cocktail and 1x with PBS only, and then eluted with GSH elution buffer (3 mg/ml reduced glutathione, 10% glycerol, 1 mM DTT). Purified GST-FBXO2 in GSH elution buffer was kindly provided by Kevin Glenn (University of Iowa). Purified protein was quantified against BSA standards by in-gel Coomassie Brilliant Blue total protein stain. *In vitro* deubiquitination reactions were carried out at 37°C with 1 µM GST-ataxin-3 (3UIM, 2UIM, or C14A) and 250 nM ubiquitin chains in 50 mM HEPES pH 7.5, 500 µM EDTA, 100 ng/ml ovalbumin, and 1 mM DTT. Reactions were stopped by the addition of 1x Laemmli buffer plus 100 mM DTT, and stored on ice until SDS-PAGE analysis. Twelve microliters of each reaction was fractionated on a 5–20% gradient gel and a 15% acrylamide gel, and analyzed by silver stain (Silver Stain Plus, Cat # 161-0449, BioRad) and Western blot analysis using P4D1 anti-ubiquitin mAb, respectively.

### Ubiquitin-AMC assays

Ubiquitin-7-amino-4-methylcoumarin (Ub-AMC, Boston Biochem Cat # U-550), GST-ATXN3(Q22)3UIM, GST-ATXN3(Q22)2UIM, and GST-FBXO2 were diluted to 2x stocks in 50 mM HEPES, 0.5 mM EDTA, 0.1 mg/ml ovalbumin, 1 mM DTT, pH 7.5 and pre-warmed to 37°C for 15 minutes. At time zero, 2x stocks of pre-warmed enzyme or buffer-only control were combined with Ub-AMC to yield 500 nM GST-tagged enzyme and 500 nM Ub-AMC in a final reaction volume of 100 µl. Ub-AMC cleavage at 37°C was detected using a Wallac 1420 multilabel fluorimeter using an excitation of 355 nm and emission at 460 nm, a lamp energy of 5160, and a counting time of 0.1 s using the Normal Aperture, Top Counter setting. Differences in initial reaction velocity were assessed by two-tailed heteroscedastic Student's t-tests.

### Immunostaining of cultured cells

24 hours before harvest, cells were re-plated on rat tail collagen-coated coverslips to ensure adequate spacing of transfected cells. Cells were rinsed with ice cold PBS, fixed in 4% paraformaldehyde/PBS, and blocked in 5% normal goat serum in 0.05% TX-100/PBS for at least 30 minutes. Primary antibodies (1H9 mAb 1∶500 and Rb anti-Flag pAb (Sigma) 1∶150 in 0.05% TX-100/PBS) were applied for one hour at room temperature. Cells were washed 3x in abundant 0.05% TX-100/PBS, before incubation for 1 hour in secondary antibodies (1∶250 goat anti-mouse AlexaFluor 568 and goat anti-rabbit AlexaFluor 488 (Molecular Probes)). Cells were washed 5x in abundant 0.05% TX-100/PBS, 5 µg/ml DAPI (Sigma cat. D9564) counterstained for 10s, rinsed briefly, and prepared for mounting with the SlowFade Anti-Fade kit (Molecular Probes, Cat #S2828) using component A according to the manufacturer's recommendations. Coverslips were sealed to glass slides with Permount (Fisher Scientific, Cat # SP15). All incubations and washes were carried out at room temperature, protected from light, unless otherwise noted.

Fixed and stained cells were gated into moderate and high levels of overexpression based on fluorescence intensity at defined exposure times using the Zeiss Axiocam MRGrab software. Moderate overexpression was defined as visibility of α-Flag staining of the cytoplasm and nucleus in the live image capture window with exposure times from 500–1000 ms; representative images for these cells were captured with the following exposures (DAPI 200 ms, 1H9 15,000 ms, α-Flag pAb 1000 ms), set to linear display (gamma  = 1) with brightness and contrast levels adjusted to span the peaks of the pixel intensity histogram. High overexpression was defined as visibility of α-Flag staining of the cytoplasm and nucleus in the live image capture window at ≤500 ms; representative images for these cells were captured with the following exposure times (DAPI 200 ms, 1H9 4500 ms, α-Flag pAb 350 ms), and adjusted as above. All images were pseudocolored and overlaid in Adobe Photoshop without further adjustment. The number of puncta per cell that were brightly positive for both α-Flag and 1H9 immunostaining was counted for the first 25 cells identified in each replicate that met fluorescence gating criteria (or the maximum number of cells to meet criteria, if less than 25), and plotted in histogram form. Statistical differences between aggregation phenotypes in 2UIM vs. 3UIM ataxin-3, 2UIM vs. UIM3-mutant ataxin-3, and 3UIM vs. UIM3-mutant ataxin-3 were assessed using the Chi-squared test of independence, using frequency data binned into populations containing 0, 1–5, 6–10, or >10 puncta per cell, giving 3 degrees of freedom (df) for each comparison.

### Analysis of protein stability with cycloheximide treatment

Cos7 cells were transiently transfected with 0.4 µg unexpanded Flag-ataxin-3(Q22) constructs one day before treatment with cycloheximide. At time zero cells were either harvested immediately or grown at 37°C, 5% CO_2_ in DMEM, 10% FBS, 1% P/S plus or minus 10 µM cycloheximide, 10 µM epoxomycin, and/or 10 mM 3-methyladenine for 10 or 24 hours before harvest in 1X Laemmli buffer plus 100 mM DTT. Lysates were resolved by SDS-PAGE and analyzed by Western blot analysis using rabbit α-Flag antibody followed by Coomassie Brilliant Blue R250 total protein staining of the PVDF membrane. Flag-tagged and total protein were analyzed densitometrically using ImageJ software, after rolling ball background subtraction with a rolling ball radius of 50. Differences in normalized abundance between constructs at each time point were assessed using a two-tailed Student's t-test, assuming unequal variance. Differences in abundance of individual constructs under 24 hr degradation-rescue conditions compared to 0 hr and 24 hr + cycloheximide conditions were assessed using paired one-tailed Student's t-tests (assuming a rescue value at 24 hours less than or equal to that at time zero, and a value greater than or equal to that at 24 hr without pharmacological rescue).

## Results

### Ataxin-3 is alternatively spliced in ATXN3 YAC transgenic mouse and human brain

Because most observations of ataxin-3 alternative splicing have been made in peripherally-derived mRNA, we wanted to confirm the presence of alternative splice variants in brain. In addition to analyzing mRNA from pooled human brain tissue, we isolated and characterized mature mRNA from the brains of ataxin-3 YAC transgenic mice, which contain the full human *ATXN3* gene and thus are an ideal model in which to examine alternative splicing of both transgenic human and endogenous murine ataxin-3 transcripts. To characterize 5′ splicing of *ATXN3* mRNA independent of 3′ variation, we PCR amplified brain-derived, Q_15_-encoding YAC cDNA using primers targeting the 5′UTR/exon1 junction and exon 9 of the human *ATXN3* transcript. Multiple minor splice variants from perinatal (data not shown) and adult murine brain were detected, cloned, and sequenced (Figure 1B). Two identified 5′ variants contain frameshift-induced stop codons upstream of multiple exon junction complexes, and thus are strong candidates for nonsense mediated decay (NMD). The remaining identified minor variants excise at least one exon encoding a Josephin domain catalytic residue (indicated by asterisks in Figure 1B), and thus are not predicted to encode functional deubiquitinating enzymes (DUBs).

We also evaluated the presence of 3′ splice variation among *ATXN3* (human) and *Atxn3* (murine) transcripts, using species-specific and sequence-specific reverse primers that selectively target either the 10-exon (2UIM) or the 11-exon (3UIM) variant. Both endogenous 3′ *Atxn3* variants were detected in nontransgenic and MJD15.4 hemizygous transgenic mice (Figure 1C). This mirrored the expression pattern of human *ATXN3* variants in three sources: nonexpanded MJD15.4 transgenic mice (Figure 1C), CAG repeat-expanded MJD84.2 transgenic mice, and cDNA from pooled adult or fetal human brain tissue (Figure 1D). Although both 3′ variants were detected in whole brain samples, amplification of 11-exon transcripts consistently produced a more robust signal than did 10-exon transcripts, independent of CAG repeat length or species of origin. Because these assays are not quantitative, this is not a strict inference of relative copy number. It does, however, illustrate that 11-exon transcripts are abundant and readily amplifiable.

### 3UIM ataxin-3 is the predominant protein isoform in murine and human brain

**Figure 2:**
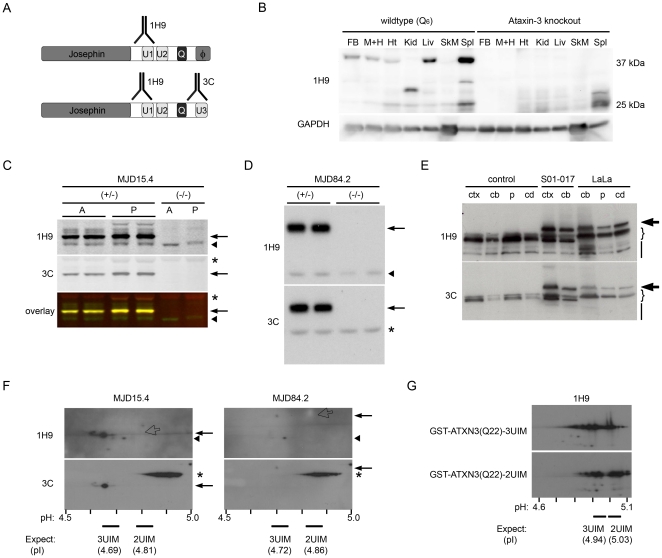
3UIM ataxin-3 is the predominant protein isoform in murine and human brain tissue. (A) Diagram of 2UIM (upper) and 3UIM (lower) ataxin-3 variants showing recognition sites for 1H9 mAb, which recognizes both isoforms, and α-ataxin-3C, which recognizes only 3UIM ataxin-3. (B) Western blot of wildtype (Q_6_) or *Atxn3* knockout mouse tissue lysates, probed for endogenous murine ataxin-3 (1H9) and GAPDH. Tissues include forebrain (FB), midbrain and hindbrain (M+H), heart (Ht), kidney (Kid), liver (Liv), skeletal muscle (SkM), and spleen (Spl). Although various putative tissue-specific splice isoforms exist, there is only one predominant isoform in brain tissue. (C–D) The major isoform (arrow) of human ataxin-3 is recognized by the 3UIM-specific antibody (3C) and 1H9 in brain tissue from hemizygous transgenic (+/−) MJD15.4 (C, Q_15_) and MJD84.2 mice (D, Q_84_), whereas endogenous Q_6_ ataxin-3 (arrowhead) is recognized only by 1H9 in hemizygous transgenic mice and wildtype (−/−) controls. Perinatal day 1–3 (P), adult (A), and non-specific 3C signal (*), as shown. (E) Both 1H9 and 3C recognize the predominant non-expanded ataxin-3 isoforms (brackets) in healthy controls and SCA3 patients (S01–017 and LaLa), as well as the predominant expanded isoform in SCA3 patients (bold arrows). Lower molecular weight bands (bars) are preferentially recognized by 1H9; cortex (ctx), cerebellum (cb), putamen (p), caudate (cd) sources, as indicated. (F,G) 2D-Western blot analysis was used to distinguish 2UIM from 3UIM ataxin-3 protein. IPG range and predicted isoelectric points (pI) of each isoform are shown. (F) In brain lysates of MJD15.4 (Q_15_)or MJD84.2 (Q_84_) YAC transgenic mice, 1H9 recognizes multiple species including endogenous murine (arrowhead) and 3UIM ataxin-3 transprotein (arrow), but does not detect any 2UIM ataxin-3 (which would be 1H9-positive, 3C-negative, 0.5 kDa larger than 3UIM ataxin-3, with a basic shift in pI, as indicated by the open arrow). The prominent band detected by 3C (*) is nonspecific. (G) 2D-Western of 50 ng purified recombinant GST-tagged ataxin-3 isoforms shows that unexpanded Q_22_ 2UIM GST-ataxin-3 is detected as readily as 3UIM GST-ataxin-3.

Neuronal toxicity in polyglutamine diseases is likely mediated primarily by the disease protein. Therefore, it was important to validate our observations of *ATXN3* splice variation at the protein level. To assess the presence and relative abundance of 2UIM and 3UIM ataxin-3 isoforms, we carried out standard and 2D Western blot analyses. The monoclonal antibody 1H9 recognizes an epitope encoded by both the 2UIM and 3UIM splice variants of human and murine ataxin-3, whereas ataxin-3C polyclonal antibody specifically recognizes human 3UIM ataxin-3 [21], as shown schematically in Figure 2A. First, to assess the range of tissue-specific isoform variation, we compared murine ataxin-3 expression in various tissues including forebrain, midbrain plus hindbrain, heart, kidney, liver, skeletal muscle, and spleen in wildtype (Q_6_) versus *Atxn3* knockout animals (Figure 2B). Endogenous ataxin-3 protein bands vary in apparent molecular weight across various tissues, consistent with the expression of tissue-specific splice variants, posttranslational proteolysis, or both. The kidney and spleen in particular contain prominent lower molecular weight isoforms distinct from the predicted 40.5 kDa full length 3UIM ataxin-3. In contrast, a single predominant isoform consistent with full length ataxin-3 is present in brain tissue. The predominant ataxin-3 bands recognized by 1H9 in standard Western blot analysis of MJD15.4 (Figure 2C), MJD84.2 (Figure 2D), and human (Figure 2E) brain lysates are also recognized by the 3UIM-specific antibody, ataxin-3C pAb. This result confirms that the 3UIM isoform of ataxin-3 is present in brain tissue from all of these sources, but is not conclusive evidence of relative abundance without further analysis, as included below.

Multiple synonymous, non-synonymous, and non-coding single nucleotide polymorphisms have been documented in the *ATXN3* gene [11], [27]. Both MJD15.4 and MJD84.2 mouse lines were constructed with YACs possessing a non-synonymous tyrosine-encoding SNP rather than the stop codon seen in the MJD1a ataxin-3 isoform. This tyrosine-encoding SNP produces a slightly longer 2UIM isoform from 10-exon transcripts (termed 2UIM-long; data not shown). Consequently, the difference in predicted molecular weight (MW) between 2UIM-long and 3UIM ataxin-3 isoforms is only 0.5 kDa in the unexpanded MJD15.4 (41.4 kDa vs. 41.9 kDa) and expanded MJD84.2 (50.2 kDa vs. 50.7 kDa) transgenic lines. To rule out the possibility that 2UIM ataxin-3 is actually present yet obscured by its close proximity to 3UIM ataxin-3, we took advantage of the difference in isoelectric point (pI) between these isoforms to assess their relative abundance. Whole brain lysates were resolved by 2D-PAGE followed by Western blot analysis with ataxin-3 specific antibodies (Figure 2F). A strong signal was detected for unmodified 3UIM ATXN3 with both ataxin-3C and 1H9 antibodies. Additional spots consistent with multiple phosphorylated forms of 3UIM ataxin-3 were also observed (slight increase in apparent MW with an acidic shift). 1H9 mAb exclusively detected spots consistent with endogenous murine 3UIM ataxin-3 (pI 4.69, MW of 40.5 kDa), mono-ubiquitinated 3UIM ataxin-3 (pI 4.81, MW ∼8 kDa greater than the major transprotein and detectable only in the abundantly expressing MJD15.4 brain), and putative smaller ataxin-3 splice isoforms or degradation products (apparent MW of <37 kDa).

Despite consistently detecting the 2UIM-encoding mRNA, we could not detect 2UIM ataxin-3 protein (pI 4.81, MW 41.9) in whole brain lysates. We were similarly unable to detect 2UIM ataxin-3 in cerebellar lysates from MJD84.2 mice (data not shown), arguing against a dramatic enrichment of this isoform in this selectively vulnerable brain region in SCA3. Recombinant 2UIM and 3UIM GST-ATXN3(Q22) were similarly resolved and detected by 2-D Western blot analysis (Figure 2G) or in-gel Coomassie staining (data not shown), indicating that the absence of detectable 2UIM ataxin-3 in brain lysates is not an artifact (due, for example, to preferential insolubility, aggregation, or precipitation during passive rehydration or IPG strip equilibration, or to a lack of recognition by the 1H9 mAb). We conclude that 3UIM ataxin-3 protein is the predominant isoform in the central nervous system. In contrast, 2UIM ataxin-3 is expressed at extremely low levels or in a highly restricted subpopulation of cells in the CNS, or is posttranslationally modified so as to be undetectable at the anticipated pI/MW.

### 2UIM and 3UIM ataxin-3 display similar in vitro DUB activity

In polyglutamine diseases, selective neuronal toxicity may result both from an expanded polyglutamine-dependent gain of function and from a partial loss of activity of the endogenous protein [27], [28]. Recent studies have shown that ataxin-3 is a member of the Josephin family of DUBs. In *Drosophila*, ataxin-3 suppresses the toxicity of expanded polyglutamine proteins in a manner that requires the catalytic activity of the Josephin domain [29]. *In vitro*, 3UIM ataxin-3 binds K48-linked, K63-linked, or mixed linkage chains and preferentially cleaves longer ubiquitin chains and K63 linkages within mixed linkage chains. These activities of ataxin-3 are UIM-dependent, as high affinity ubiquitin binding and cleavage specificity are lost when all three UIMs are mutated [16]. Intriguingly, while UIMs 1 and 2 are required for high affinity binding to ubiquitin chains, UIM3 is dispensable for this property and for the ability of ataxin-3 to cleave ubiquitin-aldehyde [19]. While both 2UIM and 3UIM ataxin-3 can bind polyubiquitinated proteins [30], the capacity of the third UIM to modulate ataxin-3 DUB activity has not been adequately assessed.

**Figure 3:**
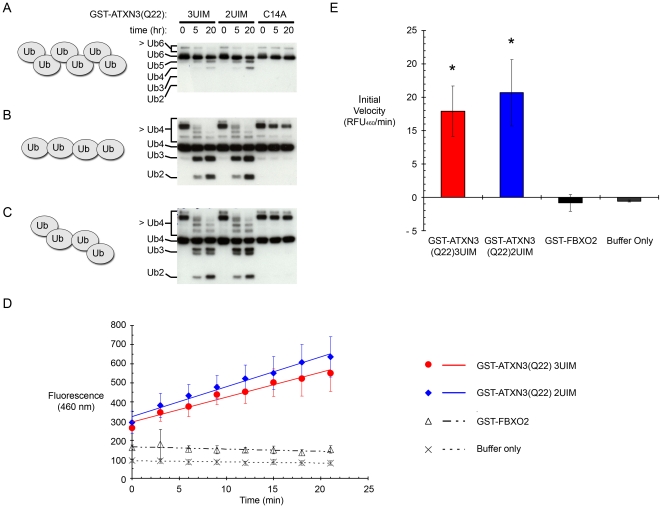
2UIM and 3UIM ataxin-3 display similar DUB activity against defined ubiquitin chains *in vitro*. (A–C) Recombinant GST-ATXN3(Q22) (3UIM or 2UIM) can cleave K48-linked hexaubiquitin (A), K63-linked tetraubiquitin (B), and mixed linkage K48-K63-K48 tetraubiquitin (C) chains. Results with catalytically inactive GST-ataxin-3 (C14A mutant) are also shown. (D–E) Recombinant 2UIM and 3UIM GST-ATXN3(Q22) cleave Ub-AMC at a similar rate. (D) Ub-AMC reaction curves. Both 3UIM and 2UIM ataxin-3 area able to cleave Ub-AMC, while reactions with either an unrelated control protein (the non-DUB F-box protein FBXO2) or buffer only show no cleavage. Error bars show standard deviations. (E) There is no significant difference between the initial reaction velocity of 2UIM and 3UIM ataxin-3(Q22) (p>0.4 by a 2 tailed heteroscedastic Student's t-test).

To test whether replacing UIM3 with the hydrophobic tail of the 2UIM isoform alters the specificity of ataxin-3 DUB activity, we incubated 1 µM purified recombinant GST- ATXN3(Q22)3UIM, GST-ATXN3(Q22)2UIM, or catalytically inactive GST-ATXN3(Q22)C14A with 250 nM defined ubiquitin chains at 37°C *in vitro*. DUB activity towards K48-hexaubiquitin, K63-tetraubiquitin, and K48-K63-K48 mixed linkage tetraubiquitin was compared by Western blot (Figure 3A–C); silver stain confirmed that equivalent levels of GST-ataxin-3 isoforms were used (data not shown). GST-ATXN3(Q22)2UIM showed the same cleavage activity as GST-ATXN3(Q22)3UIM: Limited cleavage of K48-linked Ub chains, more robust cleavage of K63-linked Ub residues and mixed linkage chains, and vigorous cleavage of the higher molecular weight Ub chains that likely represent dimers of Ub4 or Ub6 [16]. These data suggest that C-terminal splice variation does not alter the basic DUB activities of ataxin-3 and are consistent with previous reports showing the greater importance of UIMs 1 and 2 for ubiquitin-related activities of this protein, [19], [30].

To provide a more quantitative analysis of 2UIM and 3UIM ataxin-3 enzymatic function, we utilized the fluorogenic substrate Ub-AMC, which emits fluorescence at 460 nm upon cleavage of a monoubiquitin from the 7-amino-4-methylcoumarin moiety. To assess the relative ability of 2UIM and 3UIM ataxin-3 to cleave Ub-AMC, we incubated 500 nM of GST-ATXN3(Q22)3UIM or GST-ATXN3(Q22)2UIM with 500 nM of Ub-AMC. GST-FBXO2 (a recombinant GST-fusion protein that is not a DUB) and a buffer only control lacking any GST-fusion protein were used as negative controls. 2UIM and 3UIM ataxin-3 each cleaved Ub-AMC (Figure 3D). Moreover, there was no significant difference in initial reaction velocity between 2UIM and 3UIM DUB reactions (p>0.4 by a 2 tailed heteroscedastic Student's t-test). Ub-AMC was not cleaved by buffer alone or GST-FBXO2 (Figure 3E).

### 2UIM ataxin-3 is more prone to aggregate than 3UIM ataxin-3

**Figure 4:**
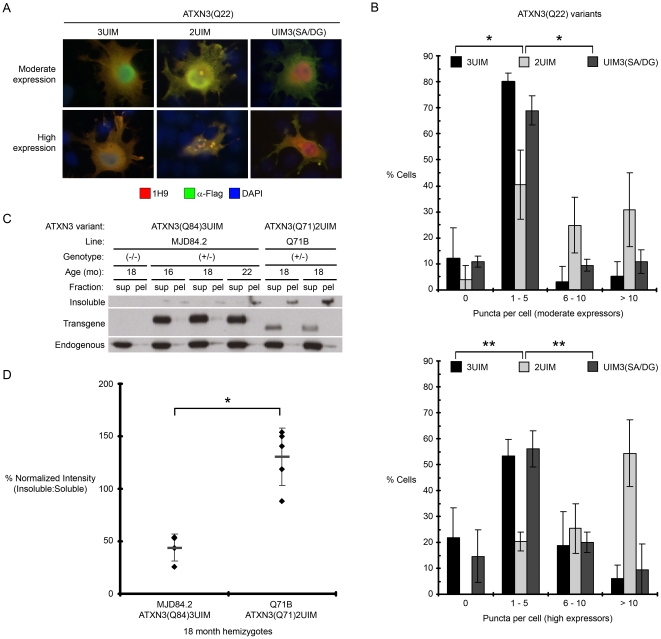
2UIM ataxin-3 is more prone to aggregate than 3UIM ataxin-3. (A) Representative immunofluorescence of Cos7 cells transiently expressing Flag-tagged ataxin-3(Q22) splice isoforms or the UIM3(SA/DG) mutant. Cells were gated by fluorescence intensity into populations of moderate and high expressors. (B) Quantification of puncta per cell in (A). Error bars represent the standard deviation within each bin. Frequency distributions differ significantly between ATXN3(Q22)2UIM and ATXN3(Q22)3UIM and between ATXN3(Q22)2UIM and ATXN3(Q22)UIM3(SA/DG) mutant ataxin-3 (*p<0.0001, ** p<1×10^-11^), but not between ATXN3(Q22)3UIM and ATXN3(Q22)UIM3(SA/DG) mutant ataxin-3 by a χ^2^ test for independence, df  = 3. (C) Supernatant (sup) and pellet (pel) fractions of non-denaturing RIPA brain lysates from aged MJD84.2 (ATXN3(Q84)3UIM) and Q71B (ATXN3(Q71)2UIM) hemizygous transgenic mice were analyzed by Western blot with 1H9 anti-ataxin-3 antibody. Insoluble microaggregates are detected at the base of lane wells, whereas soluble transprotein and endogenous ataxin-3 are visualized within the resolving gel. (D) Quantification of the ratio of insoluble to soluble ataxin-3 transprotein seen in (C). 3UIM-predominant MJD84.2 mice show a significantly lower ratio of insoluble:soluble transprotein than 2UIM-only Q71B mice (*p<0.0005 by a 1 tailed heteroscedastic Student's t-test).

While visible aggregates and inclusions may not directly cause polyglutamine-induced cytotoxicity, these pathophysiological hallmarks remain useful in identifying cells that are subject to a high burden of misfolded proteins. More subtle facets of intracellular polyglutamine protein behavior, such as alterations in subcellular localization [31], protein-protein interactions [27], and the formation of microaggregates [32], have been implicated in SCA3 toxicity. Thus, immunocytological analyses of expanded polyglutamine-expressing cells remain useful adjuncts to biochemical analyses of protein misfolding and cytotoxicity. To compare the cellular behavior of 2UIM and 3UIM ataxin-3 isoforms, we expressed Flag-tagged ataxin-3(Q22) variants with 2UIMs, 3UIMs, or 3UIMs in which the third UIM contained two mutations (A -> G, S -> D) that abolish its interactions with ubiquitin [17], [33]. This UIM3 mutant allows us to distinguish potential UIM3-specific effects from potential UIM-independent effects of substituting a hydrophobic domain (the C-terminus of 2UIM variant) for the largely hydrophilic sequence in UIM3. We transiently expressed these forms of ataxin-3 in Cos7 cells (Figure 4A and 4B) or HEK293T cells (data not shown) for 48 hours. Expression levels were confirmed by Western blot analysis with Flag and ataxin-3 antibodies (data not shown), and ataxin-3 subcellular localization was determined by immunofluorescence. Cells expressing 2UIM ataxin-3 displayed moderately robust aggregation that was not observed in cells expressing 3UIM ataxin-3 or UIM3-mutant ataxin-3. To quantify this, cells were gated by fluorescence intensity into populations of moderate or high overexpressors, and the number of immunopositive puncta per cell was counted for each ataxin-3 isoform (Figure 4b). 2UIM ataxin-3 expressing cells exhibited significantly higher aggregate formation than did 3UIM ataxin-3 or UIM3-mutant ataxin-3 expressing cells. This difference was present in moderately overexpressing cells (χ^2^ 52.6 and 22.5, respectively, df  = 3, p<0.0001) and was even more pronounced in highly overexpressing cells (χ^2^ 75.4 and 54.3, respectively, df  = 3, p<1×10^−11^). There was no difference in aggregation behavior between 3UIM and UIM3-mutant ataxin-3, whether in moderately or highly overexpressing cells (χ^2^ 5.56, df  = 3, p = 0.12 for moderate; χ^2^ 1.92, df  = 3, p = 0.59 for high overexpressors). Puncta in 2UIM ataxin-3 expressing cells generally exhibited a nuclear or nucleocytoplasmic distribution, whereas 3UIM and UIM3-mutant puncta were often exclusively cytoplasmic. In addition, puncta formed by 2UIM ataxin-3 were qualitatively larger and more irregular. Thus, in transfected cells 2UIM ataxin-3 confers an aggregation phenotype, even with a non-expanded polyglutamine domain, which does not occur with the 3UIM ataxin-3 isoform. This could be physiologically significant in cells endogenously expressing small quantities of 2UIM ataxin-3 protein, as aggregate seeding is a kinetic barrier to misfolded protein fibrillization, and nucleoplasmic ataxin-3 misfolding is critical for disease pathogenesis.

To address the possibility that this increased aggregation of 2UIM ataxin-3 reflects transient overexpression in nonneuronal cells, we compared levels of SDS-insoluble ataxin-3 aggregates in whole brain lysates from aged MJD84.2 mice, which express primarily expanded Q_84_ 3UIM ataxin-3 transprotein, and Q71B mice, which only express expanded Q_71_ 2UIM ataxin-3 transprotein (Figure 4C and 4D). Insoluble ataxin-3 aggregates are retained at the base of wells and in the stacking gel, whereas soluble (non-aggregated) human ataxin-3 transprotein and endogenous murine ataxin-3 electrophorese within the resolving gel. Given its longer repeat size, ataxin-3(Q_84_) in MJD84.2 mice might be expected to aggregate at least as readily as ataxin-3(Q_71_) expressed in Q71 B mice, provided that 2UIM and 3UIM ataxin-3 behave similarly *in vivo*. Yet at 18 months of age, 3UIM-predominant MJD84.2 YAC mice show a significantly lower ratio of aggregated (SDS-insoluble) to soluble ataxin-3 than do age-matched 2UIM-only Q71B mice (p<0.0005 by a 1 tailed heteroscedastic Student's t-test). In summary, consistent with the enhanced aggregation observed for 2UIM ataxin-3 in transfected cells, we observe more ataxin-3 aggregation in brain tissue from Q71B mice, which express only 2UIM ataxin-3, than in brain tissue from MJD84.2 mice, which express primarily 3UIM ataxin-3, despite the smaller polyglutamine expansion in Q71B mice.

### 2UIM ataxin-3 is a less stable protein than 3UIM ataxin-3 and is subject to rapid proteasomal degradation

**Figure 5:**
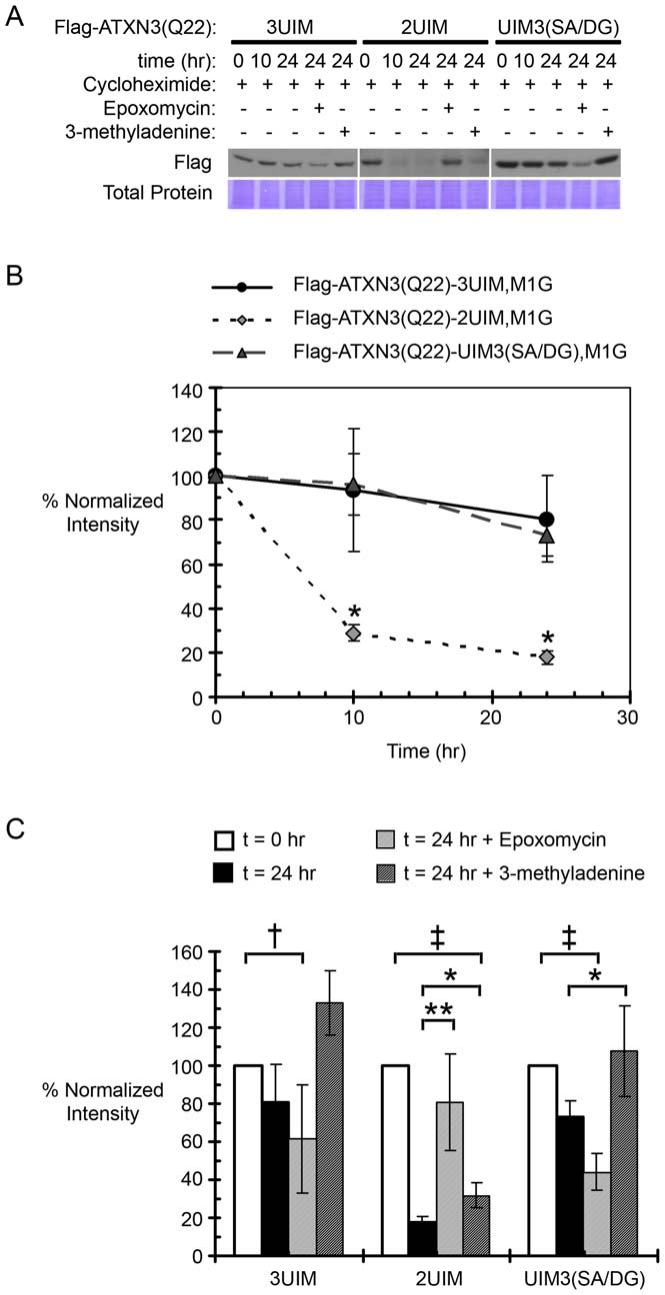
2UIM ataxin-3 is a less stable protein than 3UIM ataxin-3 and is subject to rapid proteasomal degradation. (A) Representative cycloheximide “pulse-chase” in Cos7 cells transiently transfected with Flag-tagged ataxin-3(Q22) constructs; ataxin-3 levels are visualized by anti-Flag Western blotting and total protein levels are visualized by Coomassie Brilliant Blue staining of the PVDF membrane. (B) Quantification of ataxin-3 levels during a 24 hour incubation with cycloheximide: ATXN3(Q22)2UIM is degraded significantly faster than ATXN3(Q22)3UIM or ATXN3(Q22)UIM3(SA/DG) mutant ataxin-3 at 10 and 24 hours (*p<0.02 by a two-tailed heteroscedastic Student's t-test). (C) Quantification of ataxin-3 levels during a 24 hour cycloheximide incubation in the absence or presence of the proteasomal inhibitor epoxomycin or the macroautophagy inhibitor 3-methyladenine; loss of protein at 24 hours is rescued by proteasomal inhibition for ATXN3(Q22)2UIM and by inhibition of macroautophagy for ATXN3(Q22)3UIM and ATXN3(Q22)UIM3(SA/DG) mutant ataxin-3 (†p<0.05 or ‡p<0.01 compared to time zero; *p<0.05 or **p<0.01 compared to 24 hour time point by paired one-tailed Student's t-tests). In B and C, densitometry analyses are plotted as the percentage of signal at time zero, normalized to total protein signal.

In cells, transient transfections of equivalent amounts of 2UIM and 3UIM ataxin-3 expression vectors consistently yielded lower amounts of 2UIM protein. This observation together with the enhanced aggregation propensity of 2UIM ataxin-3 prompted us to explore the relative stabilities of 2UIM and 3UIM ataxin-3 (Figure 5A and B). Cells were transiently transfected with Flag-tagged ATXN3(Q22) splice isoforms 1 day before treatment with one or more agents: 10 µM cycloheximide (CHX) to inhibit new protein synthesis, plus or minus 10 µM epoxomycin (Epox) to inhibit proteasomal degradation, or 10 mM 3-methyladenine (3-MA) to inhibit macroautophagy. At 0, 10, and 24 hours after cycloheximide addition, cells were harvested for Western blot analysis. Densitometry was used to quantify differences in the rate of protein degradation, and at each time point the Flag ataxin-3 signal, normalized to total protein, was expressed as a percentage of the normalized Flag ataxin-3 signal at time zero. In the presence of cycloheximide, 2UIM ataxin-3 levels decreased much more rapidly than did 3UIM ataxin-3, regardless of whether 3UIM ataxin-3 had a functionally intact UIM3 (*p<0.02 by a two-tailed heteroscedastic Student's t-test).

Epoxomycin and 3-MA were used to evaluate whether the proteasome, macroautophagy, or both, contributed to the degradation of unexpanded Q_22_ 2UIM versus 3UIM ataxin-3 isoforms (Figure 5C). Degradation of 2UIM ataxin-3 was nearly completely prevented by proteasomal inhibition (n.s. vs. t = 0+CHX; p<0.01 vs. t = 24+CHX), and only slightly by inhibition of macroautophagy (p<0.01 vs. t = 0+CHX; p<0.05 vs. t = 24+CHX). In contrast, proteasomal inhibition did not significantly prevent degradation of 3UIM or UIM3-mutant ataxin-3 (p<0.05 vs. t = 0+CHX; n.s. vs. t = 24+CHX), whereas inhibition of macroautophagy had an effect. Degradation of UIM3-mutant ataxin-3 was significantly prevented by 3-MA (n.s. vs. t = 0+CHX; p<0.05 vs. t = 24+CHX), and the prevention of 3UIM ataxin-3 degradation approached statistical significance (t = 24+CHX vs. t = 24+CHX +3-MA, p = 0.057). Though we did not anticipate seeing reduced 3UIM and UIM3-mutant ataxin-3 levels in the presence of epoxomycin, this observation is consistent with reports that epoxomycin treatment also induces autophagy [34]. In summary, these results are consistent with the transient expression and aggregation data: while 3UIM and UIM3-mutant ataxin-3 are relatively stable proteins with a low turnover rate, primarily via autophagy, 2UIM ataxin-3 is a highly unstable protein that is prone to misfolding and rapid degradation, primarily by the proteasome.

## Discussion

**Figure 6:**
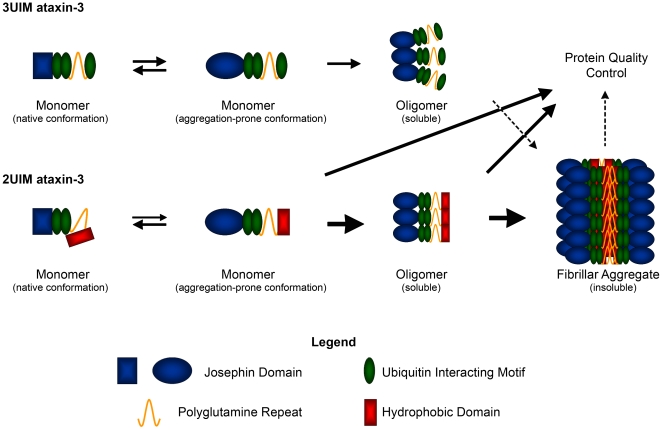
Model for the differential aggregation properties and processing of 2UIM and 3UIM ataxin-3. In the absence of polyglutamine expansion, 3UIM ataxin-3 follows a multi-domain aggregation mechanism to generate limited oligomeric species without detectable formation of SDS-insoluble fibrillar aggregates. In contrast, 2UIM ataxin-3 exists in at least two monomeric states: the native conformation, in which the hydrophobic tail remains buried and protected from the aqueous environment, and an aggregation-prone conformation with an exposed hydrophobic tail. The aggregation prone monomer can revert to the native conformation or oligomerize through both the self-association propensity of the Josephin domain (like 3UIM ataxin-3) and hydrophobic interactions of the 2UIM-specific domain. Within 2UIM oligomers, the hydrophobic C-termini will associate, increasing the local polyglutamine concentration beyond that seen in 3UIM oligomers, favoring formation of detergent-insoluble aggregates. Unstable forms of monomer and oligomer are subject to protein quality control mechanisms, including proteasomal degradation for 2UIM ataxin-3. Insoluble fibrils, which are less well handled by protein quality control systems, accumulate as biochemically and microscopically detectable aggregates.

While several minor ataxin-3 splice variants are detectable at the transcript level, we have established that full length 3UIM ataxin-3 is the major isoform expressed in brain regardless of age or polyglutamine expansion. In contrast, the originally cloned and often studied 2UIM isoform, though enzymatically similar, appears to be a highly unstable, aggregation-prone protein that is not detectable in brain tissue. Bottomley and colleagues have proposed a multi-domain model of misfolding and aggregation for ataxin-3 and other polyglutamine disease proteins [35], [36] in which early oligomer formation depends on polyglutamine-flanking sequences whereas insoluble fibrillar aggregation is polyglutamine-dependent. Building on this model, we propose that the hydrophobic C-terminus of 2UIM ataxin-3 modifies this process (Figure 6).

According to this model, 2UIM and 3UIM ataxin-3 exist in at least two monomeric states: a more stable native conformation, and an aggregation-prone conformation. For 2UIM ataxin-3, we propose that the hydrophobic C-terminus remains buried and protected from the aqueous environment in native monomers, but is exposed in the aggregation-prone conformation. To shield its hydrophobic tail from the aqueous environment, 2UIM ataxin-3 can revert to the native conformation or oligomerize through both the self-association propensity of the Josephin domain (like 3UIM ataxin-3) and intermolecular hydrophobic interactions mediated by the 2UIM-specific domain. Within 2UIM oligomers, interactions between the hydrophobic C-termini would effectively increase the local polyglutamine concentration, favoring formation of detergent-insoluble aggregates. Unstable monomers and oligomers may be degraded by the proteasome, whereas insoluble fibrils may be poorly handled by protein quality control systems and thus accumulate as biochemically and microscopically detectable aggregates. In cells and brain tissue, this process would lead to a low abundance of soluble 2UIM ataxin-3 and increased aggregation when the protein is overexpressed, as in the Q71B transgenic mouse. In more physiological 3UIM-predominant disease models, small increases in 2UIM ataxin-3 protein expression in restricted cell populations could serve as a fibrillization nidus, facilitating recruitment of expanded 3UIM ataxin-3 into toxic intranuclear microaggregates.

We confirmed the presence of multiple rare splice variants in murine brain and putative splice isoforms in other tissues. Recently, a study of 3′ *ATXN3* alternative splicing in peripheral blood leukocytes (PBL) from SCA3 patients and normal controls identified 56 distinct alternatively spliced transcripts, several of which have been previously reported or predicted [13]. While we did not detect most of these transcripts, all five minor 5′ splice variants identified by us in MJD15.4 transgenic mouse brain were also observed in non-neuronal human PBLs. Moreover, at the protein level we found that the range of putative splice isoforms detectable in leukocyte-rich splenic tissues was greater than in brain, which expresses a single, predominant full length isoform. Similar to the minor splice variants we identified in YAC transgenic brain, many PBL-derived variants are strong candidates for NMD [37], [38]. Of the remaining variants, Bettencourt et al. [13] observed that most lacked exons encoding important features of the Josephin domain, indicating that such isoforms cannot be active DUBs. Unlike our study, Bettencourt *et al.* also identified five poor candidates for NMD which encode severely truncated Josephin domains followed by frameshift-induced polyalanine repeats. We did not observe these variants in brain tissue, which argues against splicing-induced polyalanine toxicity as a major contributor to disease pathogenesis. Because all brain-derived 5′ *ATXN3* splice variants we identified were also seen in PBLs [13] and lymphocyte-rich splenic tissues, which are unaffected in human disease, they are unlikely to explain the selective neuronal toxicity observed in SCA3.

Although the 2UIM and 3UIM 3′ splice variants are both detectable in brain at the mRNA level, 3UIM ataxin-3 is clearly the predominant, physiologically relevant C-terminal splice isoform in brain. The third UIM of ataxin-3 is highly conserved across mammals and even in Xenopus, implying an important function. What that function is, however, remains elusive. Recent studies implicate the third UIM in casein kinase 2-dependent ataxin-3 phosphorylation [39], a modification that influences nucleocytoplasmic shuttling and intranuclear aggregation of ataxin-3. Consistent with this, on 2D-Western blots we observed at least two spots consistent with phosphorylated UIM ataxin-3. Together, these findings underscore the importance of further studying how the UIM3-containing C-terminus modulates ataxin-3 function within cells. They also illustrate the need for researchers to be careful when choosing among murine models when planning to study SCA3 disease pathogenesis. YAC or BAC transgenic models provide the full array of splice variants for the study of human ataxin-3 whereas existing cDNA transgenic mice only express single variants. Such models allow the study of a predominant isoform, including the ways that its behavior may be affected by the properties of minor splice isoforms. A knock-in model would be most suitable for the study of polyglutamine effects on endogenous murine ataxin-3, though no knock-in model of SCA3 yet exists.

The instability of 2UIM ataxin-3 and its propensity to aggregate suggest that while 2UIM ataxin-3 is less physiologically relevant than 3UIM ataxin-3, the 2UIM isoform may better facilitate *in vitro* studies of ataxin-3 fibrillization and aggregation or high throughput screening assays based on aggregation. In some circumstances, such as defined *in vitro* systems exploring ataxin-3 DUB activity, 2UIM ataxin-3 and 3UIM ataxin-3 behave nearly identically and will produce similar results. Nevertheless, our results demonstrate that the 2UIM and 3UIM isoforms of ataxin-3 are structurally distinct species that behave differently within the cell, and should not be used interchangeably as “full length” ataxin-3 constructs.

Because most polyglutamine disease genes are alternatively spliced and a subset of splice variants are enriched in affected neural tissues [4], [12], select alternative splicing events likely influence polyglutamine neurotoxicity. Splicing-induced differences in protein context could alter polyglutamine toxicity in numerous ways. First, increased conformational instability in the amino acid sequence flanking the polyglutamine stretch could lead to increased rates of protein misfolding and oligomerization, thereby enhancing toxicity. For example, Reid and colleagues have identified an aggregation-prone splice isoform of TBP, the disease protein in SCA17, which is enriched in its soluble form in Alzheimer disease (AD) and Huntington disease (HD) brains [7]; whether this isoform contributes significantly to SCA17 pathology or is merely a marker of dysregulated splicing in AD and HD is yet to be determined. Second, changes in protein context can alter subcellular localization; for example, functional nuclear localization signals outside of the polyglutamine domain can increase polyglutamine aggregation [40] and toxicity [31]. Finally, protein context also helps to determine protein-protein interactions, which may protect against protein misfolding or become disrupted when the polyglutamine domain is expanded, as recently described for the SCA1 disease protein, ataxin-1 [27].

Alternative splicing and other putative cell type-specific aspects of protein context are candidate determinants of selective neuronal toxicity. In addition to identifying splice variants that include or exclude polyglutamine-encoding exons, as in SCA6 [4], [41], [42], it will be important to identify any splice variants that alter physiological functions or protein-protein interactions of polyglutamine disease proteins, or that affect polyglutamine expansion-induced properties including protein misfolding and aggregation. Here we have demonstrated that alternative splicing of sequences distinct from the polyglutamine-encoding repeat can result in the production of an unstable, aggregate-prone ataxin-3 isoform, which results in increased insoluble aggregates in one murine model of SCA3. An overrepresentation of such an aggregation-prone splice variant in specific neuronal populations could contribute to the pattern of selective neuronal toxicity in one or more polyglutamine diseases.

## References

[1] Orr HT, Zoghbi HY (2007). Trinucleotide repeat disorders.. Annu Rev Neurosci.

[2] Johnson JM, Castle J, Garrett-Engele P, Kan Z, Loerch PM (2003). Genome-wide survey of human alternative pre-mRNA splicing with exon junction microarrays.. Science.

[3] Wang ET, Sandberg R, Luo S, Khrebtukova I, Zhang L (2008). Alternative isoform regulation in human tissue transcriptomes.. Nature.

[4] Tsunemi T, Ishikawa K, Jin H, Mizusawa H (2008). Cell-type-specific alternative splicing in spinocerebellar ataxia type 6.. Neuroscience Letters.

[5] Tadokoro K, Yamazaki-Inoue M, Tachibana M, Fujishiro M, Nagao K (2005). Frequent occurrence of protein isoforms with or without a single amino acid residue by subtle alternative splicing: the case of Gln in DRPLA affects subcellular localization of the products.. J Hum Genet.

[6] Sahba S, Nechiporuk A, Figueroa KP, Nechiporuk T, Pulst SM (1998). Genomic structure of the human gene for spinocerebellar ataxia type 2 (SCA2) on chromosome 12q24.1.. Genomics.

[7] Reid SJ, Whittaker DJ, Greenwood D, Snell RG (2009). A splice variant of the TATA-box binding protein encoding the polyglutamine-containing N-terminal domain that accumulates in Alzheimer's disease.. Brain Res.

[8] Paulson HL, Das SS, Crino PB, Perez MK, Patel SC (1997). Machado-Joseph disease gene product is a cytoplasmic protein widely expressed in brain.. Ann Neurol.

[9] Ichikawa Y, Goto J, Hattori M, Toyoda A, Ishii K (2001). The genomic structure and expression of MJD, the Machado-Joseph disease gene.. J Hum Genet.

[10] Hirata S, Shoda T, Kato J, Hoshi K (2003). Isoform/variant mRNAs for sex steroid hormone receptors in humans.. Trends Endocrinol Metab.

[11] Goto J, Watanabe M, Ichikawa Y, Yee SB, Ihara N (1997). Machado-Joseph disease gene products carrying different carboxyl termini.. Neurosci Res.

[12] Einum DD, Clark AM, Townsend JJ, Ptacek LJ, Fu YH (2003). A novel central nervous system-enriched spinocerebellar ataxia type 7 gene product.. Arch Neurol.

[13] Bettencourt C, Santos C, Montiel R, Costa MD, Cruz-Morales P (2010). Increased transcript diversity: novel splicing variants of Machado-Joseph Disease gene (ATXN3).. Neurogenetics.

[14] Banfi S, Servadio A, Chung MY, Kwiatkowski TJ, McCall AE (1994). Identification and characterization of the gene causing type 1 spinocerebellar ataxia.. Nat Genet.

[15] Affaitati A, de Cristofaro T, Feliciello A, Varrone S (2001). Identification of alternative splicing of spinocerebellar ataxia type 2 gene.. Gene.

[16] Winborn BJ, Travis SM, Todi SV, Scaglione KM, Xu P (2008). The deubiquitinating enzyme ataxin-3, a polyglutamine disease protein, edits Lys63 linkages in mixed linkage ubiquitin chains.. J Biol Chem.

[17] Todi SV, Winborn BJ, Scaglione KM, Blount JR, Travis SM (2009). Ubiquitination directly enhances activity of the deubiquitinating enzyme ataxin-3.. EMBO J.

[18] Schmitt I, Linden M, Khazneh H, Evert BO, Breuer P (2007). Inactivation of the mouse Atxn3 (ataxin-3) gene increases protein ubiquitination.. Biochem Biophys Res Commun.

[19] Burnett B, Li F, Pittman RN (2003). The polyglutamine neurodegenerative protein ataxin-3 binds polyubiquitylated proteins and has ubiquitin protease activity.. Hum Mol Genet.

[20] Kawaguchi Y, Okamoto T, Taniwaki M, Aizawa M, Inoue M (1994). CAG expansions in a novel gene for Machado-Joseph disease at chromosome 14q32.1.. Nat Genet.

[21] Schmidt T, Landwehrmeyer GB, Schmitt I, Trottier Y, Auburger G (1998). An isoform of ataxin-3 accumulates in the nucleus of neuronal cells in affected brain regions of SCA3 patients.. Brain Pathol.

[22] Cemal CK, Carroll CJ, Lawrence L, Lowrie MB, Ruddle P (2002). YAC transgenic mice carrying pathological alleles of the MJD1 locus exhibit a mild and slowly progressive cerebellar deficit.. Hum Mol Genet.

[23] Goti D, Katzen SM, Mez J, Kurtis N, Kiluk J (2004). A mutant ataxin-3 putative-cleavage fragment in brains of Machado-Joseph disease patients and transgenic mice is cytotoxic above a critical concentration.. J Neurosci.

[24] Todi SV, Laco MN, Winborn BJ, Travis SM, Wen HM (2007). Cellular turnover of the polyglutamine disease protein ataxin-3 is regulated by its catalytic activity.. J Biol Chem.

[25] Chai Y, Shao J, Miller VM, Williams A, Paulson HL (2002). Live-cell imaging reveals divergent intracellular dynamics of polyglutamine disease proteins and supports a sequestration model of pathogenesis.. Proc Natl Acad Sci U S A.

[26] do Carmo Costa M, Sequeiros J, Maciel P (2002). Identification of three novel polymorphisms in the MJD1 gene and study of their frequency in the Portuguese population.. J Hum Genet.

[27] Lim J, Crespo-Barreto J, Jafar-Nejad P, Bowman AB, Richman R (2008). Opposing effects of polyglutamine expansion on native protein complexes contribute to SCA1.. Nature.

[28] Matsuyama Z, Yanagisawa NK, Aoki Y, Black JL, Lennon VA (2004). Polyglutamine repeats of spinocerebellar ataxia 6 impair the cell-death-preventing effect of CaV2.1 Ca2+ channel–loss-of-function cellular model of SCA6.. Neurobiology of Disease.

[29] Warrick JM, Morabito LM, Bilen J, Gordesky-Gold B, Faust LZ (2005). Ataxin-3 suppresses polyglutamine neurodegeneration in Drosophila by a ubiquitin-associated mechanism.. Mol Cell.

[30] Berke SJ, Chai Y, Marrs GL, Wen H, Paulson HL (2005). Defining the role of ubiquitin-interacting motifs in the polyglutamine disease protein, ataxin-3.. J Biol Chem.

[31] Bichelmeier U, Schmidt T, Hubener J, Boy J, Ruttiger L (2007). Nuclear localization of ataxin-3 is required for the manifestation of symptoms in SCA3: in vivo evidence.. J Neurosci.

[32] Williams AJ, Knutson TM, Colomer Gould VF, Paulson HL (2009). In vivo suppression of polyglutamine neurotoxicity by C-terminus of Hsp70-interacting protein (CHIP) supports an aggregation model of pathogenesis.. Neurobiol Dis.

[33] Fisher RD, Wang B, Alam SL, Higginson DS, Robinson H (2003). Structure and ubiquitin binding of the ubiquitin-interacting motif.. J Biol Chem.

[34] Yang F, Yang YP, Mao CJ, Cao BY, Cai ZL (2009). Role of autophagy and proteasome degradation pathways in apoptosis of PC12 cells overexpressing human alpha-synuclein.. Neurosci Lett.

[35] Saunders HM, Bottomley SP (2009). Multi-domain misfolding: understanding the aggregation pathway of polyglutamine proteins.. Protein Eng Des Sel.

[36] Ellisdon AM, Thomas B, Bottomley SP (2006). The two-stage pathway of ataxin-3 fibrillogenesis involves a polyglutamine-independent step.. J Biol Chem.

[37] Silva AL, Romao L (2009). The mammalian nonsense-mediated mRNA decay pathway: to decay or not to decay! Which players make the decision?. FEBS Lett.

[38] Maquat LE (2005). Nonsense-mediated mRNA decay in mammals.. J Cell Sci.

[39] Mueller T, Breuer P, Schmitt I, Evert BO, Wullner U (2009). CK2-Dependent Phosphorylation Determines Cellular Localization and Stability of Ataxin-3.. Hum Mol Genet.

[40] Perez MK, Paulson HL, Pendse SJ, Saionz SJ, Bonini NM (1998). Recruitment and the role of nuclear localization in polyglutamine-mediated aggregation.. J Cell Biol.

[41] Zhuchenko O, Bailey J, Bonnen P, Ashizawa T, Stockton DW (1997). Autosomal dominant cerebellar ataxia (SCA6) associated with small polyglutamine expansions in the alpha 1A-voltage-dependent calcium channel.. Nat Genet.

[42] Kanumilli S, Tringham EW, Elizabeth Payne C, Dupere JRB, Venkateswarlu K (2006). Alternative splicing generates a smaller assortment of CaV2.1 transcripts in cerebellar Purkinje cells than in the cerebellum.. Physiol Genomics.

